# Is There a Future for Anti-CD38 Antibody Therapy in Systemic Autoimmune Diseases?

**DOI:** 10.3390/cells9010077

**Published:** 2019-12-27

**Authors:** Devis Benfaremo, Armando Gabrielli

**Affiliations:** Dipartimento di Scienze Cliniche e Molecolari, Università Politecnica delle Marche, 60126 Ancona, Italy; d.benfaremo@pm.univpm.it

**Keywords:** anti-CD38, autoimmune diseases, plasmablast, plasma cells, systemic sclerosis, SSc, systemic lupus erythematosus, SLE

## Abstract

CD38 is a type II glycoprotein highly expressed on plasmablasts, short-lived and long-lived plasma cells, but weakly expressed on other lymphoid cells, myeloid cells and non-hematopoietic cells. This expression pattern makes CD38 an interesting target for a targeted therapy aiming to deplete antibody-producing plasma cells. We present data suggesting that anti-CD38 therapy may be effective for the prevention at the preclinical stage and for the treatment of established autoimmune diseases, such as systemic lupus erythematosus, systemic sclerosis, Sjögren’s syndrome and anti-neutrophil cytoplasmic antibody (ANCA)-associated vasculitis. Given the high unmet need for efficacious disease-modifying treatment in these diseases, studies are warranted to determine if anti-CD38 antibody-based therapies may delay or prevent the disease progression of systemic autoimmune diseases.

## 1. Introduction

In the last two decades, the B cell lineage has been increasingly recognized as a major pathogenetic player in inflammation and autoimmunity [1]. Accordingly, depletion of B cells has become an attractive therapeutic target for several systemic autoimmune diseases. Indeed, B cell depletion with the anti-CD20 antibody rituximab has demonstrated to provide beneficial effects in several autoimmune disorders [2,3,4,5]. Rituximab is currently approved for ANCA-associated vasculitis, based on the results of two randomized controlled trials (RCTs) that showed its efficacy in inducing disease remission [6]. Conversely, although commonly used off-label, in systemic lupus erythematosus (SLE) and systemic sclerosis (SSc), the use of rituximab is not supported by solid evidence deriving from RCTs, but mostly derived from observational studies [7,8,9]. Moreover, in some cases, B cell-targeting agents unexpectedly resulted in the worsening of symptoms [1].

A possible explanation of the failure of such a strategy could be the fact that rituximab only depletes short-lived plasmablasts, but it does not affect the production of autoantibodies by non-proliferative long-lived plasma cells [10,11,12]. Autoantibodies are characteristic of most systemic autoimmune diseases and have an essential role in driving the diverse clinical manifestations that are observed. Therefore, a profound depletion of autoreactive plasma cells might achieve better outcomes in the treatment of these disorders. Antibodies are produced by two different compartments, short-lived plasmablasts and long-lived plasma cells. Whereas the former differentiate upon activation of B cells, the latter result from secondary immune responses and may reside in survival niches, providing the basis of the humoral side of immunological memory as well as the long-term production of autoantibodies [13]. Thus, long-lived plasma cells are preserved from the action of conventional immunosuppression or B cell depleting therapy [13]. Moreover, the depletion of B cells itself, by altering their survival niche, may foster the differentiation of short-lived into long-lived autoimmune plasma cells [14].

Bortezomib, a proteasome inhibitor approved for the treatment of multiple myeloma, was previously shown to protect mice with lupus-like disease from the development of nephritis by promoting plasma cell apoptosis through the depletion of both short-lived and long-lived subsets [1]. Furthermore, anecdotal evidence shows that bortezomib can also efficiently deplete autoantibodies and control disease manifestations in patients with various autoimmune diseases, including primary Sjögren’s syndrome, refractory SLE and ANCA-associated vasculitis [15,16,17,18,19]. Thus, the depletion of the whole plasma cell compartment might be a promising treatment option for antibody-mediated autoimmune diseases, but the unfavorable risk-benefit ratio of bortezomib may not be acceptable for patients with chronic disorders [1].

CD38 is a type II glycoprotein, involved in cell adhesion and signal transduction, highly expressed on the surface of several antibody-producing immune cells, such as plasmablasts, short lived and long-lived plasma cells, but only weakly expressed on other lineages, including lymphoid, myeloid and non-hematopoietic cells [13]. This peculiar expression pattern makes CD38 an attractive target for a treatment that aims to deplete plasma cells that produce autoantibodies.

Anti-CD38 monoclonal antibodies, such as daratumumab, have been previously demonstrated to induce a substantial depletion of plasma cells in the bone marrow of patients with refractory multiple myeloma [20,21], and are currently used in clinical practice. It is, therefore, reasonable to hypothesize that CD38 could be a potential target for the treatment of systemic autoimmune diseases by specifically depleting antibody-producing plasma cells.

We will outline below the existing evidence supporting a role of anti-CD38 targeted therapy in patients with systemic autoimmune diseases.

## 2. Evidence Supporting the Target of CD38 in Autoimmune Diseases

### 2.1. Systemic Lupus Erythematosus

Systemic lupus erythematosus (SLE) is an autoimmune disorder characterized by dysregulated immunity against the self, with abnormal production of autoantibodies that cause end-organ damage trough immune complexes deposition and chronic inflammation [22]. The occurrence of many diverse autoantibodies is a hallmark of SLE and suggests that the B cell compartment is strongly implicated in the development of the abnormal regulation of the immune response in this disease [23]. Despite recent advances in the understanding of the complexity of SLE pathogenesis, available treatment strategies fail to induce a complete remission of the disease; that, therefore, requires more effective treatment options [24].

In SLE patients, the expression of CD38 in T cells is significantly higher than in normal subjects and correlates with circulating levels of several cytokines. Additionally, increased levels of spontaneous anti-CD38 IgG autoantibodies have been observed in the sera of SLE patients with inactive disease [25]. Of note, the expression of CD38 and the levels of autoantibodies targeting this protein have an inverse correlation. Lastly, SLE patients with circulating anti-CD38 antibodies show increased plasma levels of the immunosuppressive cytokine interleukin (IL)-10. All these observations suggest that SLE patients that develop anti-CD38 antibodies have a relatively well controlled disease, which is further testified by lower SLEDAI scores and lower frequency of anti-dsDNA autoantibodies in these patients. Monocyte analyses in SLE patients showing that a higher CD38 expression in the nonclassical monocyte subpopulation is associated with a more severe disease further support the role of CD38 in SLE disease activity [26].

García-Rodríguez et al. reported that CD38 may also contribute to the inflammatory phase that precedes the development of a murine model of lupus. In this study, CD38 deficient mice showed a decreased inflammation in response to pristane administration, resulting from a reduced production of cytokines and chemokines, as well as a limitation of cell migration. Furthermore, CD38 deficiency protected mice from the onset of the disease by reducing the number of intraperitoneal apoptotic cells, which are pathogenic in this murine model of SLE. The results of this study suggest that the enzymatic activity of CD38 is important for the regulation of apoptosis in immune cells [27].

The whole B cell compartment is deranged in SLE patients. Several studies demonstrated that SLE patients show altered composition of the peripheral B cells, as well as abnormal germinal center responses and B lymphopenia. In addition to plasma cells, high levels of CD38 are also expressed by B regulatory (Breg) cells, a subset of B cells that produces IL-10 thereby promoting immune tolerance. In SLE patients, Breg cells were found to be reduced and functionally impaired [28,29], suggesting their involvement in the development and amplification of autoimmune responses.

A significant increase of antibody-producing plasmablasts, which have been associated with higher disease activity, as well as with higher titers of serum autoantibodies, is well documented in SLE [30]. Moreover, Mei et al. also demonstrated that mucosal plasmablasts are implicated in the immune response in SLE, suggesting the involvement of the mucosal immune system in SLE [30]. Likewise normal subjects, circulating plasma cells from SLE patients express higher levels of CD38 compared to other immune cells [31,32]. Lastly, using whole-genome microarray analysis, a significant elevation of the plasma cell signature was also identified in more than 30% of blood and skin specimens from SLE patients, suggesting that these methods may be useful in order to select patients with the highest likelihood of response to therapies targeted against plasma cells, including anti-CD38 antibodies [33].

In a recent study, daratumumab treatment effectively depleted the plasma cell compartment in peripheral blood mononuclear cells (PBMCs) isolated from SLE patients ex vivo [32].

The lack of effective disease-modifying treatments in SLE and the evidence of the role of CD38 positive cells in SLE pathogenesis might, therefore, encourage the use of therapies that effectively deplete antibody-producing plasma cells.

### 2.2. Systemic Sclerosis

Systemic sclerosis (SSc; scleroderma) is an autoimmune disease with a complex pathogenesis in which dysregulated immune response, vasculopathy and excessive production of collagen and extracellular matrix cooperate in the onset of abnormal fibrosis of the skin and internal organs [34]. Scleroderma is a severe and devastating disorder and progressive skin fibrosis is considered a predictor of morbidity and mortality [35]. To date, no available treatment options have been demonstrated to significantly affect the natural history of the disease [36].

Several lines of evidence indicate that the B cell compartment is strongly implicated in the pathogenesis of SSc, including autoantibody production, T cell activation, and fibrosis (reviewed in [37]). Of note, agonistic autoantibodies directed against the Platelet Derived Growth Factor Receptor (PDGFR) alpha have been observed in sera of patients with SSc [38]. These autoantibodies induce the abnormal accumulation of extracellular matrix and type I collagen trough the production of reactive oxygen species (ROS) [39]. Moreover, epitope specificity has been associated to the stimulatory or non-stimulatory nature of these autoantibodies, thereby determining their pathogenicity in vivo [40,41]. Additionally, activated B cells may contribute to the pathogenesis of SSc by promoting the differentiation of Th2 cells, thereby shifting cytokine production towards cytokines such as IL-6, IL-4, and IL-13, which in turn promote antibody production and tissue fibrosis [42].

Some studies investigated the role of antibody-producing plasma cells in SSc. In the tight-skin murine model of SSc, loss of CD19, which is also expressed by plasma cells, led to reduced autoantibody production and skin fibrosis, suggesting that CD19 signaling promotes chronic B cell activation and differentiation into autoantibody producing cells that enhance collagen deposition [43]. Like in the case of SLE, a plasma cell signature has also been identified in the skin of SSc patient as compared to healthy donors. Interestingly, the reduction of the plasma cell signature consequent to anti-CD19 treatment was highly correlated with a reduced type I collagen gene expression [33]. Not surprisingly, patients with a higher plasma cell signature at baseline showed a greater improvement in the skin score following anti-CD19 treatment than did patients without this biomarker [44].

In patients with SSc that underwent autologous hematopoietic stem cell transplantation (aHSCT), a peak in the percentage of CD38++/CD10+/IgD+ transitional B cells and CD38++/CD27++/IgD−plasmablasts has been observed already one month after the procedure. These changes persisted for several months after aHSCT and were accompanied by an increased production of IL-10 [45]. As already mentioned for SLE, it has been also observed that SSc patients show altered number and function of IL-10 producing Breg cells [46,47,48]. Moreover, in the hypochlorous acid (HOCl) murine model of SSc, additional alterations in the B cell compartment included a decrease of plasmablasts, memory B cells and Breg, with reduced IL-10 production [49]. This abnormal distribution of B cell subsets has also been constantly described in the peripheral blood of SSc patients, that show a reduced number of plasmablasts and memory B cells and an increase of naïve B cell counts [37,47,48]. Although related to a worse disease activity and severity, whether this distribution pattern is also implicated in the pathogenesis of SSc remains to be elucidated.

Although further studies are necessary to provide additional proof-of-concept evidence regarding the pathogenic role and the possible targeting of CD38, nevertheless in SSc there is an urgent need to find effective treatments that aim at restoring the deranged B cell response.

### 2.3. Sjögren’s Syndrome

Primary Sjögren’s Syndrome (SS) is a disorder characterized by reduced salivary and lacrimal flow secondary to the involvement of exocrine glands [50]. A deranged B cell response, attested by the presence of several different autoantibodies and, often, of marked hypergammaglobulinemia, is a hallmark of SS (reviewed in [51]), although compelling evidence supporting its direct role in the disease pathogenesis is currently limited [52]. Indeed, B cells targeting agents such as rituximab failed to achieve the primary endpoint (improvement of at least 30 mm in two of four visual analogue scales (VAS) by week 24) in RCTs [53].

Despite these uncertainties, a potential role of cells expressing CD38 in SS is supported by the study of peripheral blood B cells. Higher levels of mature CD38++ B cells are observed in the sera of SS patients [54], as well as a reduced number of memory B cells [55,56] and increased counts of activated T cells and plasmablasts [57]. Another recent study demonstrated that higher circulating levels of CD38+ B cells positively correlate with disease activity scores and serum levels of IgG and autoantibodies [58].

Additionally, plasma cells isolated from salivary glands of SS patients display phenotypic characteristics of the long-lived subtype, suggesting that these cells may benefit from a permissive survival niche in exocrine glands [57,59].

Overall, these data suggest that CD38+ cells are involved in the deranged immune response in SS. Therefore, treatment targeting CD38+ long-lived plasma cells may overcome the limited efficacy of anti-CD20 therapies in SS.

### 2.4. ANCA-Associated Vasculitis

Anti-neutrophil cytoplasmic antibody (ANCA)-associated vasculitis (AAV) is a group of systemic autoimmune diseases characterized by widespread neutrophil activation and vascular endothelial damage secondary to the production of ANCA autoantibodies [60]. Treatment with a B cell depleting agent, such as rituximab, combined with glucocorticoids induces clinical remission in a significant percentage of patients with AAV [61,62]. However, long-term glucocorticoid treatment is often needed to maintain remission and prevent disease flares. Therefore, to avoid excessive exposure to glucocorticoids and, more importantly, to promote a long-lasting remission of the disease, there is a need for alternative effective treatment options [6].

In peripheral blood of AAV patents, higher levels of circulating CD38+ plasma cells have been reported to significantly correlate with disease activity [63]. In addition, an increased number of circulating B cells that express CD38 is associated to a higher risk of relapse during a phase of remission [64].

Targeting CD38+ plasma cells may, therefore, represent a new reasonable treatment option to promote a long-lasting remission in patients with AAVs.

## 3. Potential Limitations of Targeting CD38 in Autoimmune Diseases

Despite the fascinating perspectives of targeting CD38 in autoimmune diseases, there are also several potential limitations that should be underlined. Most of them arise from the broader immunomodulatory role of CD38 itself, which is linked to its multifunctional activity as an ecto-enzyme [65]. In fact, CD38 is located on the surface of plasma cells and catalyzes the conversion of nicotinamide dinucleotide (NAD+) to cyclic adenosine diphosphate ribose (cADPR) via cyclase activity and cADPR to ADPR, leading to the accumulation of adenosine [66]. Since adenosine exerts an immunosuppressive activity, as observed in the bone marrow niche in multiple myeloma, targeting CD38 with daratumumab may also boost host-antitumor immune response, although these preliminary observations need to be confirmed [66,67]. While this appears to be a favorable indirect effect of daratumumab in clonal neoplastic diseases such as multiple myeloma, using a drug that promotes the activity of effector T cells in systemic autoimmune diseases may not be as beneficial [68].

Another possible limitation of using anti-CD38 antibodies in autoimmune diseases is that, as already mentioned, CD38 is also widely expressed by IL-10 producing regulatory B cells [67]. Therefore, it could be argued that the depletion of immunosuppressive and regulatory subtypes may limit the efficacy of anti-CD38 therapies, or, in worst cases, induce a flare of the autoimmune disease. Indeed, in murine models the loss of CD38 was able to induce autoimmune features such as autoantibodies and end-organ damage [69]. However, the role of CD38 in the development of autoimmunity and its implications are still controversial, as is the role of regulatory B cells in patients with established autoimmune diseases, in which they have been shown to be already reduced and functionally impaired [28,29,47,70].

Additionally, treatment with anti-CD38 antibodies would not affect some important subsets such as memory B cells and germinal center B cells, that lack this surface marker [1]. Consequently, this approach would not prevent disease relapses by activation of these specific cells [71]. Indeed, the combination of anti-CD38 and anti-CD20 therapy could overcome this issue by depleting all autoreactive clones, but potentially at the cost of more severe side effects. The possibility to use such a combination strategy in systemic autoimmune diseases is attractive but needs further investigation.

Finally, it has to be considered that a profound depletion of long-lasting antibody producing plasma cells, either by anti-CD38 alone or, more importantly, in combination with other targeted therapies that affect memory cells, could also reduce the production of protective antibodies to previous infections, such as smallpox and measles [72], thereby exposing the recipients to an increased infectious risk.

## 4. Conclusions

The data we presented support a role for anti-CD38 therapy for the treatment of systemic autoimmune diseases, such as SLE, SSc, SS and AAV, not only in established disease, but also at the preclinical stage. Given the important need to find effective disease-modifying therapies in these diseases, it could be important to develop targeted therapies stratifying patients based on plasma cell and plasmablast signature analysis. Further studies are needed to properly assess whether anti-CD38 antibody-based therapies could be beneficial in systemic autoimmune diseases.
